# Lead Levels in Wild Boar Meat Sauce (Ragù) Sold on the Italian Market

**DOI:** 10.3390/ijerph18083989

**Published:** 2021-04-10

**Authors:** Antonio Lenti, Alessandro Menozzi, Giorgio Fedrizzi, Simonetta Menotta, Tiziano Iemmi, Giorgio Galletti, Paolo Serventi, Simone Bertini

**Affiliations:** 1 Department of Veterinary Science, University of Parma, Strada Del Taglio 10, 43126 Parma, Italy; antonio.lenti@unipr.it (A.L.); tiziano.iemmi@unipr.it (T.I.); paolo.serventi@unipr.it (P.S.); simone.bertini@unipr.it (S.B.); 2 Istituto Zooprofilattico Sperimentale della Lombardia e dell’Emilia Romagna, Reparto Chimico degli Alimenti–Via Pietro Fiorini 5, 40127 Bologna, Italy; giorgio.fedrizzi@izsler.it (G.F.); simonetta.menotta@izsler.it (S.M.); giorgio.galletti@izsler.it (G.G.)

**Keywords:** lead, wild boar, ragù, meat, ICP-MS

## Abstract

Game meat is endowed with excellent nutritional value, but it may also be a possible source of harmful substances, such as mycotoxins and heavy metals. In particular, several studies showed that lead fragments from hunting ammunition are able to represent a residual contaminant in the meat of wild boars or deer, representing a possible source of lead absorption. Even though wild boar meat consumption in Italy is rather limited, this meat could also be present in very popular Italian recipes, such as the typical meat sauce called ragù. We evaluated the lead levels in 48 samples (three different batches for each of the 16 brands) of ready-to-eat wild boar meat ragù sold on the Italian market in food stores and online distribution with the inductively coupled plasma-mass spectrometry (ICP-MS) technique. A high variability was found in the lead levels detected in the samples, with a median lead level of 0.10 mg/kg (0.01–18.3 mg/kg) and some of the samples showing very high lead concentrations. Since no intake level of lead is considered completely safe, and maximum levels for game meat have so far not been established, a greater attention on the risks to consumers’ health related to the presence of this heavy metal in game meat is recommended.

## 1. Introduction

Game meat is acknowledged to possess a higher nutritional value [1] compared to the meat of farm animals; as a matter of fact, the meat of wild animals tends to have a lower fat content and a higher percentage of proteins with respect to the meat of farmed species [2]. This is probably, at least in part, due to the fact that game animals are more active and live in a natural environment, feeding with their natural diet. Moreover, game meat generally has a very desirable fatty acid (FA) composition, with a high content of beneficial omega-3 polyunsaturated FA and is a good source of essential elements [2]. However, even though there has been an overall increasing request by consumers of meat products for animals bred in more natural environments, game meat consumption in Europe remains rather low, since a small percentage of the population (about 2–4%), mainly constituted by hunters and their relatives, eats this type of food with regularity [3]. Together with the much lower availability on the market, one of the reasons that could discourage the average consumer from eating game meat products is the fear of a higher risk that wild animal meat may be contaminated by harmful substances, such as mycotoxins, pesticides, and heavy metals. Indeed, the lack of control on the food consumed by wild animals, together with the fact that maximum safe levels for most contaminants in game meat have not been set by public health authorities, may represent a potential danger for the consumers, and one not to be underestimated. Game meat is an excellent source of beneficial essential metals, such as iron and zinc, but can also contain potentially harmful levels of toxic heavy metals, such as lead, cadmium, mercury, and arsenic. Indeed, in the meat of lead-shot game, such as deer [4,5], birds [6], and wild boars [7], high levels of lead, and a huge presence of lead fragments can be found. Although the toxicity of lead is well known, and its use has been progressively dismissed, it is still widely employed for the production of ammunition, batteries, weights, and radiation shielding. Lead damages primarily the nervous system, but it can also harm the kidneys [8]; the bones [9]; and the cardiovascular [10], immune, and reproductive systems [11,12].

Lead is also suspected to be carcinogenic to humans, and inorganic lead compounds are listed in the 2A group by the International Agency for Research on Cancer (IARC), [13]. In addition, children are extremely sensitive to lead toxicity since even very low doses are able to hinder nervous system development, causing permanent cognitive deficits and serious behavioural changes [14,15]; therefore, no blood level of this metal is considered safe for them.

The European Commission has established a maximum accepted lead level of 0.10 mg/kg in the meat of cattle, pig, lamb, and chicken [16], but until now, a maximum safe level of this metal in game meat has not been established, and this may represent a possible risk for the consumers’ health.

In a previous study by our group, we found the presence of a massive dispersion of lead bullet fragments in the carcasses of hunted wild boars in Italy, together with a high concentration of lead in meat samples [7]. Even though the risks for human health may be mitigated by the limited consumption of this type of meat, there are also some very popular Italian dishes that may contain wild boar meat. For example, pasta dressed with a typical meat sauce recipe called “ragù” is a very commonly consumed food in Italy, and especially in some regions, the ragù made with wild boar meat is very appreciated, either home-made using fresh meat or purchased as a ready-to-eat product. Another aspect to be considered is that meat processing and cooking techniques used for the production of Italian ragù could play a role in promoting lead poisoning; for instance, marinating and long cooking times with vinegar, wine, lemon juice, or other acid substances may enhance lead absorption because of the complexation of the metal with acidic ligands [17].

The aim of the present study was, therefore, to assess the lead levels of ready-to-eat wild boar meat ragù sold in Italy, by means of inductively coupled plasma-mass spectrometry (ICP-MS) analysis, with the ultimate goal to protect the consumer’s health.

## 2. Materials and Methods

### 2.1. Samples Collection

Forty-eight samples belonging to 16 different brands (identified as A, B, C, … P) of wild boar meat ragù sold on the Italian market (food stores and online distribution) were collected and analyzed for lead concentration by means of the ICP-MS technique. Lead levels were assessed in three different batches for each brand (identified as A1, A2, A3, and so on), collected between March and November 2020, in order to acquire a more thorough knowledge of the presence of the heavy metal in the food sold by different producers. The meat used for the production of ragù was processed in three different establishments located in three different Italian regions: Emilia-Romagna (*n* = 15 samples), Toscana (*n* = 27), and Trentino Alto-Adige (*n* = 6 samples).

Wild boar ragù assayed in this study is sold in glass jar containers of 170–180 g product, and all the brands are provided with CE marking, authorizing the free commercialization of the food on the EU market. The percentage of wild boar meat in the product sold by different brands was variable, ranging from 15% to 60% (mean value 48.4 ± 14.8%).

After the collection, all the samples were kept at room temperature until analysis, according to the storage instructions reported on the product labels.

### 2.2. Lead Level Analysis

High purity deionized water was obtained from Evoqua Water Technologies (Barbsbüttel, Germany); nitric acid was purchased from J.T. Baker (Center Valley, PA, USA); hydrochloric acid was from Sigma-Aldrich (St. Louis, MO, USA).

Each sample was homogenized, and two subsamples (weighing 3 ± 0.02 g) were obtained, put in screw cap polypropylene sample tubes (50 mL Digi-Tubes, SCP Science, Montreal, Canada), and 10 mL of nitric acid were added; the samples were then placed in a Digi-Prep system (SCP Science, Montreal, Canada) at 75 °C overnight. After cooling, the samples were diluted to a final volume of 200 mL with an acid solution and analyzed by means of ICP-MS (7700 Agilent Technologies, Santa Clara, CA, USA) with an ASX-500 CETAC Autosampler (Cetac Technologies, Omaha, NE, USA).

The analytical method has been previously validated according to international regulations (Reg. 333/2007/EU and Reg. 882/2004/EU Annex 3). The method validation was performed, evaluating for Pb linearity (solvent and matrix), limit of quantification, specificity, precision (under repeatability conditions), trueness, and reproducibility (intra-laboratory).

Linearity was performed analyzing standard concentrations from 0.1 to 100 µg/L (six concentrations—five replicates/concentration) and blank matrices (muscle) fortified with defined Pb concentrations from 0.003 to 2 mg/kg (ten concentrations—six replicates/concentration). Limit of quantification was evaluated at least 10 times over the signal of blank sample (0.005 mg/kg for each metal). Specificity was performed by analyzing negative samples (*n* = 20) and by verifying the absence of inferences. Precision was calculated by fortifying samples at three different levels (six replicates per level) in the validation range and evaluating repeatability as coefficient of variation (CV < 10%). Trueness was evaluated by the recovery percentage calculated on the same samples (>80% for Pb). Reproducibility (CV < 15%) was evaluated on the same samples at the same concentrations but repeating the analysis three different times during a month. Uncertainty (from 20 to 22%) was calculated with “in house data” using bottom-up approach (Ellison and Williams 2012). The method was continuously monitored by analysis of Community Bureau of Reference (BCR) samples in every batch and evaluating that the parameters were within the predicted values. The laboratory employed BCR-185R (bovine liver) with a 0.172 mg/kg Pb concentration and a defined uncertainty of 0.009 mg/kg (0.0163–0.181 mg/kg) and participated in proficiency tests organized by international committees such as FAPAS and European Union reference laboratories (EURL).

### 2.3. Statistical Analysis 

Statistical analysis was performed by means of GraphPad Prism ver.7 software (GraphPad Software Inc., La Jolla, CA, USA). All data were tested for normality by means of the Kolmogorov–Smirnov test. The results were expressed as median and range or as mean ± SD, according to the presence or absence of a normal distribution. Mann–Whitney *U* Test was employed to evaluate the significance of the difference among groups of data. Linear regression analysis and Spearman correlation coefficient analysis were used to assess the correlation between lead concentrations and the percentage of wild boar meat in the sample. A value of *p* < 0.05 was considered statistically significant.

## 3. Results

The results of the lead level analysis on all the samples are reported in Table 1.

The median value of lead concentration detected in all the samples was 0.10 mg/kg (0.01–18.3 mg/kg). In 23 samples out of 48 (47.9%), lead levels above 0.10 mg/kg were measured. The highest lead levels were detected in samples L2, B3, K1, O1, and E2 (1.30, 1.60, 5.80, 9.00, and 18.3 mg/kg, respectively). In particular, the samples with a higher percentage of wild boar meat (>50%) showed median lead levels of 0.07 mg/kg (0.01–9.00 mg/kg), while in those with a lower amount of wild boar meat (35–50% and <35%), median lead concentrations of 0.10 mg/kg (0.03–18.3 mg/kg) and 0.16 mg/kg (0.09–1.30 mg/kg), respectively, were measured.

**Figure 1:**
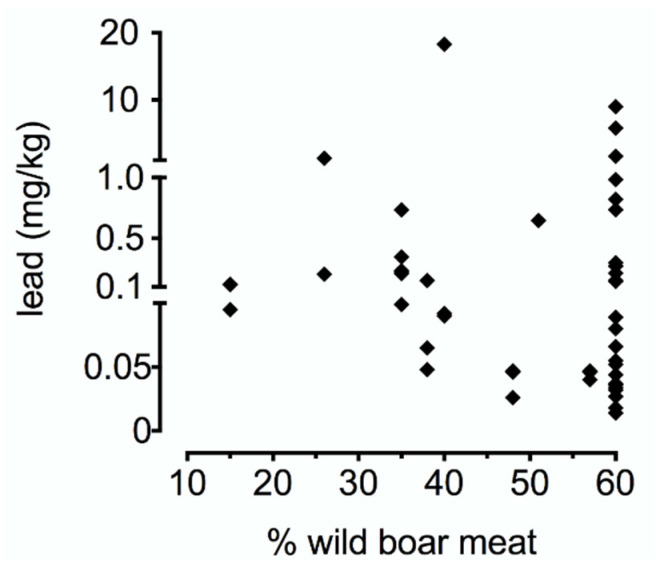
Correlation between the percentage of wild boar meat in ragù samples and lead levels.

No significant correlation was found between the percentage of wild boar meat in the samples and the lead concentrations (r = −0.18, *p* = 0.21) (Figure 1). 

In addition, no significant differences were found comparing lead levels measured in the samples of the different brands.

No significant differences in lead levels were found according to the geographic localization of the meat processing establishments. Median lead levels detected in ragù samples processed in Emilia-Romagna, Toscana, and Trentino Alto-Adige were, respectively: 0.05 mg/kg (0.01–9.00), 0.15 mg/kg (0.03–5.80), and 0.09 (0.05–18.3), *p* = 0.32 (data not shown).

## 4. Discussion

The results of this study evidenced that, in a high percentage of assessed samples of Italian ragù made with wild boar meat, a lead concentration above 0.10 mg/kg (the maximum accepted level in the EU for the meat of most farm animals) was detected. A high variability was found in the heavy metal levels measured in the meat sauce samples, since lead levels ranged from 0.01 to 18.3 mg/kg; a high variability was also found among the lead levels detected in different batch samples of the same brand. For example, sample E2 had the highest lead concentration (18.3 mg/kg), but the other two batch samples (E1 and E3) belonging to this brand showed considerably lower levels of the metal (0.09 mg/kg). In a similar manner, in one of the three batch samples of brand K and O, much higher lead levels were detected with respect to the other two.

This great variability of lead concentrations in the wild boar ragù samples is unlikely to be explainable by an environmental contamination and is instead suggestive of a random presence of lead fragments derived from ammunition in the meat of the hunted ungulates. In accordance with this observation, no differences were found comparing lead levels measured in ragù samples processed in game handling centres of the different Italian regions, suggesting that the geographical origin of the wild boars is unlikely to have a relevant influence on the concentrations of the heavy metal in the final product.

Several studies have already shown that, in big size game, lead bullets are subjected to a huge fragmentation due to the impact and passage through the body of the animals, and hundreds of tiny fragments are able to radiate into tissues up to 45 cm away from the bullet trajectory [4,18,19]. Indeed, the analysis of lead levels in muscles of hunted wild boars showed a huge variability according to the absence or presence of metal shards of different size in the assessed samples [7,20].

This high level of dispersion of tiny lead parts in the carcasses of the hunted animals makes it very difficult to perform a thorough finishing in order to exclude contaminated meat from human consumption since there is also the formation of nanoparticles that are not removable from edible tissues [21]. Indeed, previous studies showed that, in packages of processed meat of hunted deer, 59% [18] and 80% [4] of the tested samples contained lead fragments. Moreover, it was shown that in pigs fed for two days with this venison meat, lead blood levels were four times higher (2.29 μg/dL) compared to pigs fed with a control diet (0.63 μg/dL) [4]. In addition, ingested bullet lead fragments were sometimes retained in parts of the large intestine with the result of enhancing the heavy metal absorption, and of causing gut diseases [22,23]. 

Even though some samples in our study showed very high lead levels, and the mean concentration of this toxic metal was found to be more than nine times higher than the maximum safe level fixed for meat products, it might be difficult to assess if these data could represent a real threat for the health of the consumers. Moreover, the type of food assessed in this study contains, in addition to wild boar meat, several other ingredients such as vegetables and/or swine and bovine meat in variable proportions, which may also contribute to the total lead level measured in the sample, thus it might be difficult to extrapolate the level of lead contamination due to wild boar meat. Additionally, the percentage of wild boar meat employed in the recipes by the different producers is also variable, and this could further complicate the interpretation of lead levels; these may therefore be considered as limitations of the present study.

As a matter of fact, there is still no consensus among the scientific community about whether the presence of bullet-derived lead in the meat of game destined for human consumption represents an actual hazard for the health of the consumers. While some concluded that the risk for human health might be considered negligible [24,25], several other authors have highlighted an increased risk due to lead exposure, at least for people who regularly eat game meat [4,26,27].

There seems to be general agreement, however, on the fact that there is probably no “safe” threshold intake for lead in humans [28] and, accordingly, the provisional total weekly intake of 25 µg/kg previously established by the World Health Organization was withdrawn in 2011 on the basis of toxicological studies on human infants and adults [14,29,30,31,32].

In 2010, the German Federal Institute for Risk Assessment carried out an assessment of the risk associated with consumption of game meat, which showed evidence of a possible health risk for assiduous consumers of game meat (i.e., hunters and their relatives/friends). Furthermore, in various research works, it has been observed that the consumption of fresh meat of game shot with traditional lead-based hunting ammunition resulted in a significantly higher lead intake with respect to meat from animals killed with lead-free ammunition, and that this may result in a potential enhanced risk for human health [27,33].

Thus, in the evaluation of the risk related to lead contamination of game meat, the existence of a part of the population with a possible very high level of exposure must be considered, despite the fact that this food represents only a minimal component of the diet of the average population [34]. However, it has been estimated that about 1% of the EU population may be frequent consumers of lead-shot game meat and that tens of thousands of children in the EU may be consuming such lead-contaminated meat often enough to cause impairment of cognitive development [34]. 

In Italy, it has been estimated that 3% of the Italian population are regular consumers of wild game meat, with an average weekly consumption of 100–200 g meat for adults, and about 50 g for children [35,36]. With regard to Italy, it should be also pointed out that meat sauce is a frequently consumed food throughout the country, and the availability of wild boar ragù in food stores and online distribution could expose a wide part of the population, not necessarily consisting of hunters or assiduous consumers of fresh game meat, to lead contamination.

The use of non-lead bullets, in combination with more effective game meat hygienic measures, are, therefore, possible strategies to be employed [33] in order to ensure a more thorough protection of human health. However, a more accurate process of discarding the meat close to wound channels may cause significant economic losses without granting a complete removal of lead particles from the meat. On the other side, the increased cost for the hunters due to the use of more expensive lead-free ammunition could be balanced by the opportunity of selling safer game meat, free from bullet-derived lead, to a larger number of consumers.

## 5. Conclusions

In conclusion, the results of this study showed evidence that samples of ragù made with wild boar meat sold on the Italian market may be contaminated by very high levels of the heavy metal, up to 18.3 mg/kg, possibly related to the presence of bullet fragments in the meat used for food production. Since no intake level of lead is, at present, considered completely devoid of risks for human health, and some people are assiduous consumers of meat with a high probability of substantial lead contamination, a more thorough legislation about the maximum concentrations of this metal in foodstuffs, including game meat, may be recommendable.

## Figures and Tables

**Table 1:** Lead levels (mg/kg) detected in wild boar ragù samples.

Sample	Lead	Lead (Mean ± SD)	% Wild Boar Meat
A1	0.05	0.05 ± 0.01	57%
A2	0.05		
A3	0.04		
B1	0.15	0.61 ± 0.86	60%
B2	0.09		
B3	1.60		
C1	0.03	0.02 ± 0.01	60%
C2	0.02		
C3	0.01		
D1	0.06	0.09 ± 0.06	60%
D2	0.05		
D3	0.15		
E1	0.09	6.16 ± 10.5	40%
E2	18.3		
E3	0.09		
F1	0.05	0.09 ± 0.05	38%
F2	0.15		
F3	0.07		
G1	0.73	0.39 ± 0.29	35%
G2	0.23		
G3	0.21		
H1	0.21	0.17 ± 0.13	60%
H2	0.27		
H3	0.03		
I1	0.04	0.13 ± 0.15	60%
I2	0.04		
I3	0.30		
J1	0.10	0.23 ± 0.13	35%
J2	0.35		
J3	0.23		
K1	5.80	2.22 ± 3.13	60%
K2	0.82		
K3	0.04		
L1	0.21	0.72 ± 0.55	26%
L2	1.30		
L3	0.65		
M1	0.03	0.04 ± 0.01	48%
M2	0.05		
M3	0.05		
N1	0.03	0.06 ± 0.03	60%
N2	0.08		
N3	0.07		
O1	9.00	3.57 ± 4.70	60%
O2	0.74		
O3	0.98		
P1	0.12	0.11 ± 0.01	15%
P2	0.12		
P3	0.10		

Total median lead level: 0.10 mg/kg (0.01–18.3 mg/kg).

## Data Availability

Raw data are available on request addressed to corresponding author.
